# Effect of Fluid Flow Rate on Efficacy of Fluid Warmer: An In Vitro Experimental Study

**DOI:** 10.1155/2018/8792125

**Published:** 2018-07-08

**Authors:** Vorasruang Thongsukh, Chanida Kositratana, Aree Jandonpai

**Affiliations:** Department of Anesthesiology, Faculty of Medicine Ramathibodi Hospital, Mahidol University, Bangkok 10400, Thailand

## Abstract

**Introduction:**

In patients who require a massive intraoperative transfusion, cold fluid or blood transfusion can cause hypothermia and potential adverse effects. One method by which to prevent hypothermia in these patients is to warm the intravenous fluid before infusion. The aim of this study was to determine the effect of the fluid flow rate on the efficacy of a fluid warmer.

**Methods:**

The room air temperature was controlled at 24°C. Normal saline at room temperature was used for the experiment. The fluid was connected to an infusion pump and covered with a heater line, which constantly maintained the temperature at 42°C. The fluid temperature after warming was measured by an insulated thermistor at different fluid flow rates (100, 300, 600, 900, and 1200 mL/h) and compared with the fluid temperature before warming. Effective warming was defined as an outlet fluid temperature of >32°C.

**Results:**

The room temperature was 23.6°C ± 0.9°C. The fluid temperature before warming was 24.95°C ± 0.5°C. The outlet temperature was significantly higher after warming at all flow rates (*p* < 0.001). The increases in temperature were 10.9°C ± 0.1°C, 11.5°C ± 0.1°C, 10.2°C ± 0.1°C, 10.1°C ± 0.7°C, and 8.4°C ± 0.2°C at flow rates of 100, 300, 600, 900, and 1200 mL/h, respectively. The changes in temperature among all different flow rates were statistically significant (*p* < 0.001). The outlet temperature was >32°C at all flow rates.

**Conclusions:**

The efficacy of fluid warming was inversely associated with the increase in flow rate. The outlet temperature was <42°C at fluid flow rates of 100 to 1200 mL/h. However, all outlet temperatures reached >32°C, indicating effective maintenance of the core body temperature by infusion of warm fluid.

## 1. Introduction

The normal core body temperature in humans is maintained by the hypothalamus and typically ranges from 36.5°C to 37.5°C. The interthreshold range is usually only 0.2°C to 0.4°C. Both general and regional anesthesia inhibit central thermoregulation and increase the interthreshold range to 2°C to 4°C, leading to hypothermia [1, 2]. Hypothermia is defined as a decrease in the core body temperature to <36°C [1]. The combination of hypothermia, acidosis, and coagulopathy has been identified as the “lethal triad” in trauma patients because it increases the risk of morbidity [3]. Intraoperative hypothermia is associated with postoperative myocardial ischemia, impaired coagulation, an increased risk of wound infection, and atrioventricular arrhythmia [3, 4]. Massive cold fluid or blood infusion is one cause of hypothermia. One liter of fluid at room temperature will reduce the mean body temperature by approximately 0.25°C [5]. Induction of mild hypothermia without extracorporeal circulation, a 30-min infusion of 2 L of normal saline at 4°C, decreases the core body temperature by 2.5°C [6]. The theoretical impact of fluid infusion on the body temperature can be calculated as follows:

(1) $$\begin{array}{c} \text{change}\;\;\mathrm{in}\;\;\mathrm{mean}\;\;\mathrm{body}\;\;\mathrm{temperature}=\frac{\text{thermal}\;\;\mathrm{stress}\;\;\mathrm{of}\;\;\mathrm{infused}\;\;\mathrm{fluids}}{\left(\text{weight}\times \text{sp}\;\;\mathrm{heat}\right)}, \end{array}$$

where thermal stress = difference between core body temperature and temperature of infused fluid (°C) × specific heat of infused fluid × volume of infused fluid (L/h), weight = weight of patient (kg), and sp heat = specific heat of patient (0.83 kcal/L/°C). Sp heat of infused fluid: blood, 0.87 kcal/L/°C; saline, 1 kcal/L/°C [7, 8].

According to this equation, the temperature of the infused fluid should not be <36°C in a normothermic patient to prevent a decrease in the mean body temperature during anesthesia. Jung et al. [9] developed a fluid warming device and suggested that effective warming was indicated by a warmed fluid temperature of >32°C. According to the above equation, when infusing fluid at 32°C at a flow rate of 2 L/h to a normothermic patient whose core body temperature is 36°C and body weight is 50 kg, the mean body temperature will decrease by only 0.2°C.

Warm intravenous infusion is a direct, active method of warming the core body temperature and is suitable for patients who require massive transfusion [10]. The temperature of the warmed intravenous fluid should not exceed 40°C to 42°C to avoid denaturation of plasma proteins [11]. The aim of this experimental study was to determine the effect of the infusion rate on the temperature of warmed fluid. Very high flow rate may cause ineffective fluid warming due to short transit time. The hypothesis was that increasing the infusion rate may decrease the efficacy of warming by the heat line. The fluid temperature before warming was the control variable, the different infusion rates were independent variables, and the difference in the fluid temperature after warming was the dependent variable.

## 2. Methods

**Figure 1:**
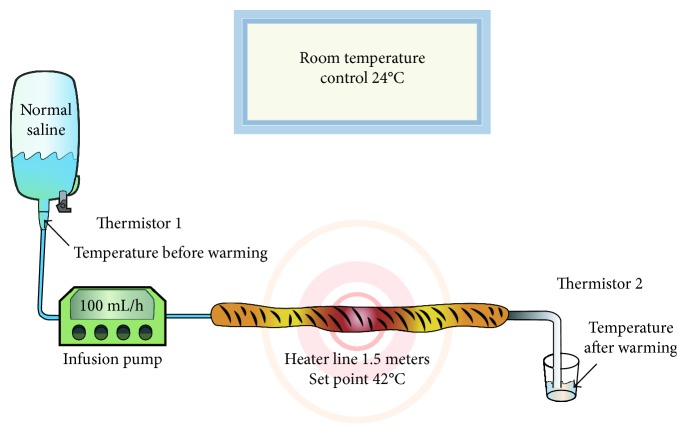
Schematic diagram of the experimental set-up.

This experimental study was performed in the general operating room at Ramathibodi Hospital. The ambient temperature was controlled at 24°C via a room air conditioning system which was close to the operating condition in our institution. Normal saline was used in this study because it is commonly used as an isotonic solution for fluid replacement in operating rooms. The fluid was maintained at the temperature of the operating room, then connected to the infusion pump (Terofusion; Terumo Europe, Leuven, Belgium), which was adjusted to flow rates of 100, 300, 600, 900, and 1200 mL/h, and these ranges of fluid flow rate were commonly infused in major abdominal operation in local setting. A 1.5 m heater line (Barkey autocontrol 3XPT; Barkey, Leopoldshöhe, Germany) covered the infusion line. The heater line was set at 42°C. After rinsing the warmed fluid for 5 min at each flow rate to maintain a constant temperature after warming, we recorded the room temperature by the electronic thermometer and the temperature of the fluid before and after passing through the heater line. Each flow rate was studied five times. The outlet temperature was measured at the immediate end of the heater line with an insulated digital thermistor (Life Scope i; Nihon Kohden, Tokyo, Japan) sealed in a 4 mL test tube. Before the study, the reliability and accuracy of thermistor were checked by repeatedly measuring the neonatal incubator temperature. Effective warming was defined as an outlet temperature of >32°C [9]. The experiment and data collection were completely done in one day. The schematic diagram is depicted in Figure 1.

Statistical analyses were performed using SPSS v.20.0 for Windows (IBM SPSS Inc., Armonk, NY), and a data chart was produced using Microsoft Excel 2007. All fluid temperatures are reported as mean ± standard deviation. The normality assumption for continuous variables was assessed by the Shapiro–Wilk test. A paired *t*-test or Wilcoxon's test was used to assess the statistical significance of the changes in temperature before and after warming, and an independent *t*-test or the Mann–Whitney *U* test was used to assess the changes in temperature between each flow rate and the next higher one. All statistical analyses were two-sided with a significance level at *p* value < 0.05.

## 3. Results

**Figure 2:**
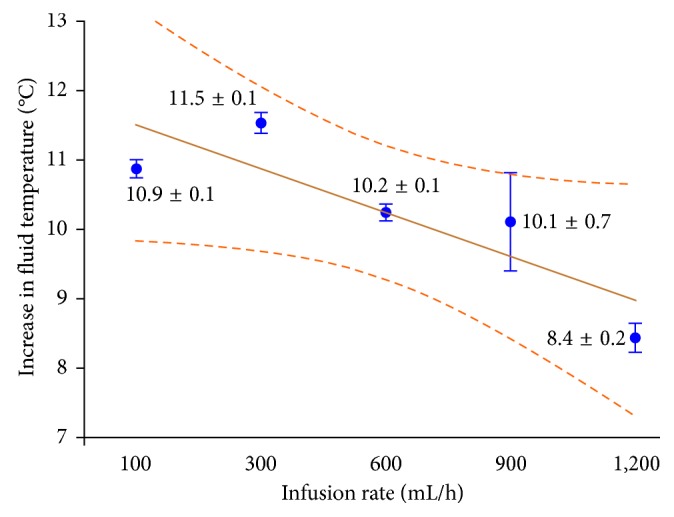
Increasing temperatures after warming at different infusion rates.

The room temperature during the experiment was 23.6°C ± 0.9°C. The temperature of the fluid before warming was 24.9°C ± 0.5°C. The outlet temperature significantly increased after warming at all infusion rates (*p* < 0.001). The maximum outlet temperature was 36.9°C at a flow rate of 300 mL/h. The fluid temperature increased by 10.9°C ± 0.1°C, 11.5°C ± 0.1°C, 10.2°C ± 0.1°C, 10.1°C ± 0.7°C, and 8.4°C ± 0.2°C at flow rates of 100, 300, 600, 900, and 1200 mL/h, respectively (Table 1 and Figure 2). For all flow rates except 300 mL/h, the outlet temperature did not reach the ideal temperature of 36°C; however, all of the outlet temperatures were >32°C. The change in temperature was significantly different among all flow rates (*p* < 0.001) except the change between 600 and 900 mL/h. A linear trend was found for the entire set of flow rates (as shown in Figure 2), suggesting that the effectiveness of warming tended to be inversely correlated with the increase in flow rate.

## 4. Discussion

One of the main factors that affected the warming capacity in this experimental study was the ambient temperature. The operative room air conditioning system caused an ambient temperature fluctuation of 23.6°C ± 0.9°C, leading to variation in the fluid temperature before warming. However, the recommended operating room temperature that can prevent perioperative hypothermia ranges from 20°C to 24°C, which was within our range [12, 13]. The initial temperature of the fluid before warming is 24.9°C ± 0.5°C, higher than the room temperature may be from the different location of measurement and not well distributed of airflow. The heater line was constantly maintained at 42°C, but the outlet temperature at each flow rate was <42°C. Only the outlet temperature at a flow rate of 300 mL/h was >36°C. Nevertheless, the outlet temperature at all flow rates was >32°C, indicating effective warming. With respect to the warming efficacy of other devices, the Hotline (Smiths Medical, St. Paul, MN) can warm the fluid to 34.8°C at a flow rate of 80 mL/h, and the Astotherm (Armstrong Medical, Coleraine, Northern Ireland) can warm the fluid to 30°C at the same flow rate [7]. In clinical practice, infusion of warmed fluid more effectively maintains the patient's core body temperature than does infusion of room temperature fluid [14].

In the present study, the efficacy of fluid warming decreased as the flow rate increased. The change in temperature was dependent upon the duration of time that the venous line was in contact with the heater line for heat exchange. Therefore, the fluid can be completely heated at lower flow rates. However, we found that the efficacy of warming was lower at a flow rate of 100 than 300 mL/h; this may have been due to the higher rate of heat dispersal after warming to the atmosphere in the temperature measurement technique.

Based on our study, fluid warming with the heater line (Barkey autocontrol 3XPT®, Germany) can effectively increase infused fluid temperature for replacement therapy in uneventful abdominal operation. However, further research is required to see if more rapid flow rate can still generate optimal temperature or not. It is also worthwhile to compare the performance of some traditional fluid warming devices because some devices can effectively warm the fluid at higher flow rate than in our study.

## 5. Conclusion

Intravenous fluid warming is an alternative warming method by which to prevent hypothermia in patients who required a massive fluid transfusion. In the present study, the effectiveness of warming was inversely associated with the increase in the flow rate. Although the outlet temperature was <42°C as the set point of the heater line, all tested flow rates (100–1,200 mL/h) were able to increase the outlet temperature to >32°C, which was considered to be effective fluid warming fluid.

## Figures and Tables

**Table 1:** Fluid temperature before and after warming.

Infusion rate (mL/h)	Fluid temperature before warming (°C)	Fluid temperature after warming (°C)	Difference in fluid temperature (°C)	*p* value
100	25.0 ± 0.1	35.9 ± 0.1	10.9 ± 0.1	<0.001^*∗*^
300	24.8 ± 0.1	36.3 ± 0.2	11.5 ± 0.1	<0.001^*∗*^
600	25.0 ± 0.2	35.2 ± 0.1	10.2 ± 0.1	<0.001^*∗*^
900	24.1 ± 0.7	34.2 ± 0.0	10.1 ± 0.7	<0.001^*∗*^
1,200	24.5 ± 0.2	33.0 ± 0.2	8.4 ± 0.2	<0.001^*∗*^

Data are presented as mean ± standard deviation. ^*∗*^Statistically significant at *p* < 0.001.

## Data Availability

The datasets generated and/or analyzed during the current study are available from the corresponding author. The authors will personally share the data with academic institutions upon a reasonable request.

## Additional Points

*Limitations*. The main limitation of the present study is that by its inherent nature, it cannot be extrapolated to the clinical setting. The effectiveness of the warming device and flow rate cannot represent the actual core body temperature after infusion of warmed fluid. However, the findings of this study will serve as a baseline for future clinical studies. Lee et al. [15] suggested that both the flow rate and length of the catheter from the warmer outlet to the patient influence the effectiveness of the warmer. A longer catheter may be associated with more heat loss to the ambient; thus, the outlet fluid should be located as close as possible to the patient.

## Abbreviations

Sp heat: Specific heat.

## References

[1] Sessler D. I. (2008). Temperature monitoring and perioperative thermoregulation. *Anesthesiology*.

[2] Butterworth J. F., Mackey D. C., Wasnick J. D., Butterworth J., Mackey D. C., Wasnick J. (2013). Thermoregulation hypothermia, & malignant hyperthermia. *Morgan and Mikhail’s Clinical Anesthesiology*.

[3] Moore E. E. (1996). Thomas G. Orr Memorial Lecture. Staged laparotomy for the hypothermia, acidosis, and coagulopathy syndrome. *American Journal of Surgery*.

[4] Tsuei B. J., Kearney P. A. (2004). Hypothermia in the trauma patient. *Injury*.

[5] Sessler D. I., Miller R. D. (2005). Temperature monitoring. *Miller’s Anesthesia*.

[6] Rajek A., Greif R., Sessler D. I., Baumgardner J., Laciny S., Bastanmehr H. (2000). Core cooling by central venous infusion of ice-cold (4 degrees C and 20 degrees C) fluid: isolation of core and peripheral thermal compartments. *Anesthesiology*.

[7] Smith C. E., Karl W. (2008). Principles of fluid and blood warming in trauma. *ITACCS*.

[8] Horowitz P. E., Delagarza M. A., Pulaski J. J., Smith R. A. (2004). Flow rates and warming efficacy with Hotline and Ranger blood/fluid warmers. *Anesthesia and Analgesia*.

[9] Jung S. W., Han T. H., Lee J. Y. (2006). Performance characteristics of high efficiency fluid and blood warmer using print circuit board heater at various flow rates. *Korean Journal of Anesthesiology*.

[10] Kumar S., Wong P. F., Melling A. C., Leaper D. J. (2005). Effects of perioperative hypothermia and warming in surgical practice. *International Wound Journal*.

[11] Vazquez R., Larson D. F. (2013). Plasma protein denaturation with graded heat exposure. *Perfusion*.

[12] AORN Recommended Practices Committee (2007). Recommended practices for the prevention of unplanned perioperative hypothermia. *AORN Journal*.

[13] Pennsylvania Patient Safety Advisory Group (2008). Prevention of inadvertent perioperative hypothermia. *Patient Safety Authority*.

[14] Smith C. E., Geddes E., Swede S. (1998). Warming intravenous fluids reduces perioperative hypothermia in women undergoing ambulatory gynecology surgery. *Anesthesia and Analgesia*.

[15] Lee S. H., Kim H. K., Park S. C., Kim E. S., Kim T. K., Kim C. S. (2010). The effect of infusion rate and catheter length on the temperature of warming fluid. *Korean Journal of Anesthesiology*.

